# Clinical Validation of the Aptima Bacterial Vaginosis and Aptima *Candida/Trichomonas* Vaginitis Assays: Results from a Prospective Multicenter Clinical Study

**DOI:** 10.1128/JCM.01643-19

**Published:** 2020-01-28

**Authors:** Jane R. Schwebke, Stephanie N. Taylor, Ronald Ackerman, Robert Schlaberg, Neil B. Quigley, Charlotte A. Gaydos, Steven E. Chavoustie, Paul Nyirjesy, Carmelle V. Remillard, Philip Estes, Byron McKinney, Damon K. Getman, Craig Clark

**Affiliations:** a University of Alabama at Birmingham, Birmingham, Alabama, USA; b Louisiana State University Health Sciences Center, New Orleans, Louisiana, USA; c Comprehensive Clinical Trials, West Palm Beach, Florida, USA; d University of Utah, Salt Lake City, Utah, USA; e Geneuity CRS, Maryville, Tennessee, USA; f Johns Hopkins University, Baltimore, Maryland, USA; g Segal Trials, Miami, Florida, USA; h Drexel University, Philadelphia, Pennsylvania, USA; i Hologic, Inc., San Diego, California, USA; Brigham and Women's Hospital

**Keywords:** bacterial vaginosis, candidiasis, trichomoniasis, Nugent score, Amsel criteria, molecular test, diagnostic accuracy, sensitivity, specificity, clinician’s diagnosis, Aptima

## Abstract

Infectious vaginitis due to bacterial vaginosis (BV), vulvovaginal candidiasis (VVC), and Trichomonas vaginalis accounts for a significant proportion of all gynecologic visits in the United States. A prospective multicenter clinical study was conducted to validate the performance of two new *in vitro* diagnostic transcription-mediated amplification nucleic acid amplification tests (NAATs) for diagnosis of BV, VVC, and trichomoniasis. Patient- and clinician-collected vaginal-swab samples obtained from women with symptoms of vaginitis were tested with the Aptima BV and Aptima *Candida/Trichomonas* vaginitis (CV/TV) assays. The results were compared to Nugent (plus Amsel for intermediate Nugent) scores for BV, *Candida* cultures and DNA sequencing for VVC, and a composite of NAAT and culture for T. vaginalis. The prevalences of infection were similar for clinician- and patient-collected samples: 49% for BV, 29% for VVC due to the *Candida* species group, 4% for VVC due to Candida glabrata, and 10% for T. vaginalis. Sensitivity and specificity estimates for the investigational tests in clinician-collected samples were 95.0% and 89.6%, respectively, for BV; 91.7% and 94.9% for the *Candida* species group; 84.7% and 99.1% for C. glabrata; and 96.5% and 95.1% for T. vaginalis. Sensitivities and specificities were similar in patient-collected samples. In a secondary analysis, clinicians’ diagnoses, in-clinic assessments, and investigational-assay results were compared to gold standard reference methods. Overall, the investigational assays had higher sensitivity and specificity than clinicians’ diagnoses and in-clinic assessments, indicating that the investigational assays were more predictive of infection than traditional diagnostic methods. These results provide clinical-efficacy evidence for two *in vitro* diagnostic NAATs that can detect the main causes of vaginitis.

## Abstract (precis)

Infectious vaginitis due to bacterial vaginosis (BV), vulvovaginal candidiasis (VVC), and Trichomonas vaginalis accounts for a significant proportion of all gynecologic visits in the United States. A prospective multicenter clinical study was conducted to validate the performance of two new *in vitro* diagnostic transcription-mediated amplification nucleic acid amplification tests (NAATs) for diagnosis of BV, VVC, and trichomoniasis.

## INTRODUCTION

Vaginitis is responsible for as many as 50% of all gynecologic visits in the United States and represents a major contributor to health care expenses (1). Infectious vaginitis due to bacterial vaginosis (BV), vulvovaginal candidiasis (VVC), and trichomoniasis accounts for up to 90% of these cases (2). Unlike trichomoniasis, both BV and VVC are attributable to several pathogens. For VVC, overgrowth of Candia albicans is predominant, although other *Candida* species, including Candida glabrata, may contribute as well (3). BV is harder to diagnose because the pathogenesis involves decreased levels of *Lactobacillus* bacteria concomitant with increased concentrations of BV-associated bacteria, such as Gardnerella vaginalis, *Mobiluncus* spp., and Atopobium vaginae (4, 5).

Various diagnostic methods are available to identify the underlying cause of vaginitis. In the clinician’s office, a combination of pH, a potassium hydroxide (KOH) test, and microscopic examination of fresh samples of vaginal discharge are routinely used, despite their relatively poor performance (1, 6). For BV, diagnosis often relies on the use of either clinical Amsel criteria or Gram stain and Nugent score (considered the gold standard laboratory method for diagnosis of BV). Examination of wet mounts with KOH preparation and/or vaginal cultures for *Candida* are the most common diagnostic tools for VVC. Highly sensitive and specific nucleic acid amplification tests (NAATs) are recommended for detecting Trichomonas vaginalis, but examination of wet-mount preparations is still commonly used in clinical practice (6). However, several barriers are associated with the use of nonmolecular methods, including lack of equipment in the clinic, subjectivity of the clinical endpoints used and inconsistent employment between practitioners, lack of proper training in microscopy, and overall poor sensitivity of the tests (7–9). Diagnosis of the underlying cause of vaginitis is further complicated by the common symptomatology reported for BV, VVC, and trichomoniasis (2, 6); the incidence of mixed infections or coinfections (10–12); and the recurrence of vaginal symptoms (13–16).

Taken together, these barriers result in many women being misdiagnosed based on nonspecific observations, leading to incorrect, misguided, or prolonged treatment (7, 9). This article reports the results from the clinical validation of two newly developed U.S. Food and Drug Administration (FDA)-cleared NAATs for the detection of infectious vaginitis. The study also compares the investigational-assay results, in-clinic testing results, and clinicians’ diagnoses to reference methods for BV, the *Candida* species group, C. glabrata, and T. vaginalis.

## MATERIALS AND METHODS

### Study design and ethics approval.

A multicenter cross-sectional diagnostic-accuracy study was conducted to evaluate the clinical performance of two NAATs, the Aptima BV and Aptima *Candida/Trichomonas* vaginitis (CV/TV) assays. The studies were conducted in accordance with the ethical principles derived from the Declaration of Helsinki and the Belmont Report and in compliance with the FDA and Good Clinical Practice Guidelines set forth by the International Conference on Harmonization (ICH-E6). The study protocols were approved by the local institutional review board at every site. Written informed consent was obtained from each subject at the time of enrollment, prior to specimen collection. Participants were compensated for study participation.

### Study population.

Subjects at least 14 years of age with symptoms of vaginitis (e.g., abnormal vaginal discharge, vaginal odor, genital itching or irritation, pain/discomfort during sexual intercourse or urination, edema, or erythema) were eligible for enrollment. Subjects were enrolled at 21 U.S. sites (clinical research centers and emergency medicine, family planning, public health, sexually transmitted infection [STI], and family medicine/obstetric-gynecologic clinics) between June and October 2018. Exclusion criteria included use of douches, vaginal deodorants, or intravaginal products within 48 h of enrollment or prior enrollment in the study.

For each subject, the collection site provided subject demographic and clinical data, including the following: clinicians’ diagnoses and subject-reported date of birth, sex, ethnicity, race, symptoms of STIs, pregnancy status, menstrual status, recent (i.e., within 24 h) unprotected sexual intercourse, HIV diagnosis, history of recurrent symptoms of vaginitis within 12 months, and use of feminine products within 4 weeks.

### Sample collection.

**FIG 1:**
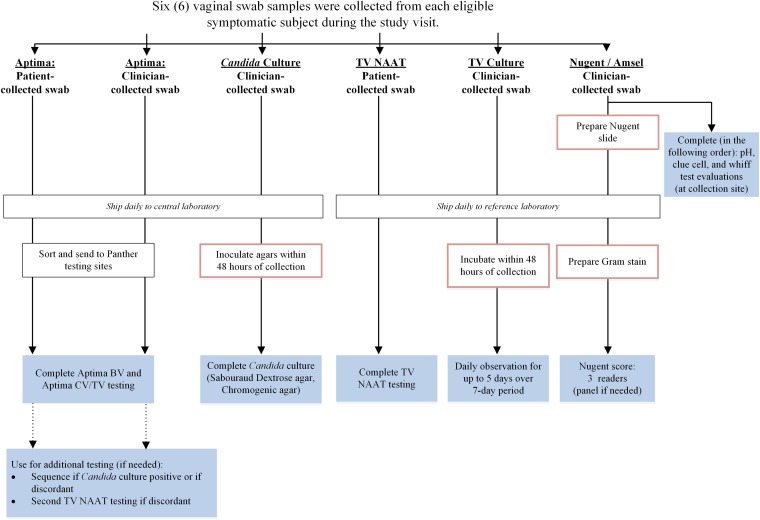
Sample workflow and testing algorithm. The red boxes denote sample preparation or handling. The shaded boxes denote testing for each swab.

Six vaginal-swab samples were collected in the clinic from each patient during routine clinical visits (Fig. 1) using a predetermined rotating order: two investigational-assay swabs (one patient collected and one clinician collected) (Aptima multitest swab; Hologic, Inc., San Diego, CA), one patient-collected swab for T. vaginalis NAAT (Xpert vaginal/endocervical swab; Cepheid, Sunnyvale, CA), one clinician-collected swab for *Candida* sp. culture (BD BBL CultureSwab EZ; Becton, Dickinson and Company, Sparks, MD), and one clinician-collected cotton swab each for Nugent score/Amsel criteria and T. vaginalis culture. All swab samples were collected according to package insert directions and instructions for use for each collection device.

Each investigational swab was tested with both the Aptima BV and Aptima CV/TV assays on the Panther system (Hologic, Inc., San Diego, CA). Paired investigational-swab samples were split evenly among three U.S. laboratories. The Aptima BV assay reported positive or negative results for BV based on a mathematical algorithm analysis of rRNA detection of *Lactobacillus* species, G. vaginalis, and A. vaginae. The Aptima CV/TV assay reported positive or negative results based on the detection of RNAs for (i) the *Candida* species group (C. albicans, Candida dubliniensis, Candida parapsilosis, and Candida tropicalis), (ii) C. glabrata, and (iii) T. vaginalis.

For BV, the reference method was comprised of a consensus Nugent score and modified Amsel criteria, if necessary. Using modified Amsel criteria, the presence of ≥20% clue cells, together with either a vaginal pH greater than 4.5 (from chromatic paper touched to the swab) or a positive whiff test (a potassium hydroxide test on the swab), indicated BV infection. For each subject, a single vaginal swab was first smeared on a glass microscope slide to prepare the Nugent scoring slide and then used to complete Amsel evaluation. The slide was then Gram stained and assigned a Nugent score based on the relative combined concentration of Gram-positive rods (e.g., *Lactobacillus* spp.), Gram-variable/negative rods and cocci (e.g. G. vaginalis and *Bacteroides* spp.), and curved Gram-variable rods (e.g., *Mobiluncus* spp.) characteristic of bacterial vaginosis (17). The sum of the three components was calculated for the final Nugent score and interpretation; using Nugent criteria, a sample with a total score of 7 or more was considered BV positive, a score of 3 or less was considered BV negative, and a score of 4 to 6 was considered intermediate. Each Gram-stained slide was independently reviewed by three different reviewers at a single reference laboratory who were blind to each other’s interpretations. If the BV interpretations (positive, negative, or intermediate) of at least two reviewers agreed, consensus was reached, and the Nugent interpretation was final. Disagreement across all three reviewers was resolved via panel review of the same slide at a multihead microscope. If the final consensus Nugent interpretation was intermediate, modified Amsel criteria, which excluded abnormal discharge, were used to determine the BV status.

For candidiasis, the reference method was comprised of yeast culture and PCR/bidirectional sequencing for a subset of specimens. For each subject, a single vaginal swab was used to inoculate two different culture media at a single reference laboratory: Sabouraud dextrose agar and CHROMagar Candida (CHROMagar, Paris, France), both read after 48 h. The growth level on both media was reported as no colony, 1+, 2+, 3+, and 4+ (*n*+ represented the number of quadrants showing *Candida* growth). For subjects with a positive culture result on either medium, identification of the isolated yeast species was performed by bidirectional sequencing of the *its2* gene (18) using both clinician- and patient-collected Aptima vaginal-swab samples; an isolated *Candida* species was determined to be present if it was detected on either swab.

For T. vaginalis, the reference method was comprised of the Xpert TV assay (Cepheid, Sunnyvale, CA) and the InPouch TV culture system (Biomed Diagnostics, Inc., White City, OR). The Xpert TV assay swab sample from each subject was tested at a single reference laboratory. For each subject, one cotton swab was used to inoculate the InPouch TV system at the clinical site; each inoculated pouch was incubated within 48 h at a single reference laboratory, and readings were performed daily for up to 7 days. If either test was positive for a subject, the subject’s status was “infected”; both reference tests had to be negative to establish a “noninfected” status. Aptima swab samples left over after Aptima CV/TV assay testing were tested with the Aptima Trichomonas vaginalis assay in the case of discordance between the investigational assay result and the T. vaginalis infection status.

### In-clinic assessments.

For this study, the following in-clinic testing was performed and documented for vaginitis. For BV, Amsel criteria were assessed individually (pH of >4.5, clue cells comprising ≥20% of total cells, or a positive whiff test) or as a group with (original Amsel) or without (modified Amsel) abnormal vaginal discharge factored in. For candidiasis, KOH test results were assessed. Results from standard in-clinic testing for T. vaginalis, such as wet-mount microscopy, were not documented for this study; InPouch culture performed at a central laboratory was used as a surrogate for in-clinic testing.

### Clinician diagnosis.

Diagnosis was based on the clinician’s assessment of in-clinic testing or other standard of care testing (e.g., wet mount for T. vaginalis or detection of pseudohyphae for *Candida*), clinical signs, patient-reported symptoms, and subject history.

### Statistical methods.

Prevalence, sensitivity, specificity, positive predictive value, and negative predictive value were calculated according to standard equations. Analyses were performed separately for clinician- and patient-collected Aptima swab samples. The confidence intervals for sensitivity and specificity were calculated using the score method (19). The confidence intervals for positive and negative predictive values were calculated based on exact confidence intervals for the ratio of two independent binomial proportions (20). Samples with inconclusive reference results and samples with invalid or missing investigational-assay results were excluded from the analyses. Analyses were performed with SAS software (version 9.4; SAS Institute Inc., Cary, NC).

## RESULTS

### Study design and subject accountability.

There were 1,519 subjects enrolled for participation in both studies. Of these, 17 subjects were withdrawn for various reasons, including ineligibility to participate or self-termination of participation. An additional 85 subjects were excluded from the Aptima BV analysis (a Nugent score was not evaluable [*n* = 58] or available [*n* = 26] or an intermediate Nugent score could not be resolved [*n* = 1]), and 6 subjects were excluded from the Aptima CV/TV analysis (withdrawn reference samples [*n* = 4], invalid Aptima CV/TV results for both swabs [*n* = 1], or no swabs collected [*n* = 1]). A total of 1,501 subjects were evaluable for BV and/or CV/TV and were included in the evaluable study population; 1,417 subjects were evaluable for BV and 1,496 for CV/TV in at least one sample type.

Table 1 provides summarized patient-reported demographics and clinical characteristics of the evaluable study population. Most subjects were between 18 and 39 years of age (69.4%), black or African American (50.2%), from southeast or southwest U.S. clinical centers (66.0%), and enrolled at clinical research centers or obstetrics and gynecology clinics (75.3%). Most of the subjects were not pregnant (98.2%), were of reproductive age but not actively menstruating (81.5%), or had not been diagnosed with HIV (97.7%). The most frequently reported symptoms were abnormal vaginal discharge (81.9%); vaginal odor (56.8%); or genital itching, irritation, or soreness (62.2%). Almost 60% of the evaluable subjects had a history of recurrent symptoms of vaginitis within the previous 12 months. A minority of the subjects (20.3%) reported the use of feminine products (e.g., douches, tampons, or deodorants) within 4 weeks of collection.

**TABLE 1:** Demographic and clinical characteristics of evaluable subjects

Category	No. of patients (%)
Age (yr)^*a*^	
14–17	5 (0.3)
18–29	554 (36.9)
30–39	485 (32.3)
40–49	247 (16.5)
≥50	210 (14.0)
Race/ethnicity	
Asian	74 (4.9)
Black/African American	753 (50.2)
White (Hispanic/Latino)	269 (17.9)
White (not Hispanic/Latino)	341 (22.7)
Other^*b*^	64 (4.3)
Geographic region	
Mid-Atlantic	244 (16.3)
Northeast	220 (14.7)
Northwest	49 (3.3)
Southeast	571 (38.0)
Southwest	417 (27.8)
Site type	
Clinical research center	659 (43.9)
Family planning clinic	282 (18.8)
Hospital system high-risk STI clinic	18 (1.2)
Obstetrics and gynecology clinic	467 (31.1)
Public health clinic	75 (5.0)
Subject-reported symptoms^*c*^	
Abnormal vaginal discharge	1,230 (81.9)
Vaginal odor	853 (56.8)
Genital itching/irritation/burning/soreness	933 (62.2)
Pain during sex and/or urination	371 (24.7)
Edema	127 (8.5)
Erythema	138 (9.2)
Other	30 (2.0)
Pregnant	
Yes	21 (1.4)
No	1,474 (98.2)
Unspecified	6 (0.4)
Menstrual status	
With menses	120 (8.0)
Without menses	1,224 (81.5)
Postmenopausal	157 (10.5)
Diagnosed with HIV	
Yes	14 (0.9)
No	1,467 (97.7)
Unspecified	20 (1.3)
History of recurrent symptoms within 12 mo	
No	623 (41.5)
Yes	874 (58.2)
1 or 2 occurrences	396 (45.9)
3 or 4 occurrences	242 (28.1)
>4 occurrences	224 (26.0)
Unknown	4 (0.3)
Use of feminine products within 4 wk	
Yes	305 (20.3)

a The median age was 33.0 years (minimum, 14 years; maximum, 79 years). The mean age was 35.3 years ± 11.74 years (standard deviation).

b Includes patient-reported other, mixed, and unknown races.

c May report multiple responses.

### Infection rates.

Rates of single and multiple infections by BV-associated organisms, the *Candida* species group, C. glabrata, and T. vaginalis are shown in Table 2, based on detection in samples tested by reference methods or in clinician- and patient-collected samples tested with the investigational assays. Overall, the infection rates identified by the reference methods and the investigational assays were similar. Using reference methods, BV alone was detected in 31.9% of the total subjects, the *Candida* species group alone in 13.9%, C. glabrata alone in 0.9%, trichomoniasis alone in 1.8%, and no infection in 31.1%. Infections rates were similar for the investigational assays in clinician- and patient-collected swabs. Coinfection rates by 2 or more organisms were 20% by reference testing and approximately 25% by investigational testing.

**TABLE 2:** Single- and multiple-infection rates determined by clinical reference method and investigational NAAT

Infection^*a*^	Result [no. (%)]^*b*^		
	Reference method (*n* = 1,365)	Investigational NAAT	
		Clinician collected (*n* = 1,491)	Patient collected (*n* = 1,480)
All negative	425 (31.1)	430 (28.8)	399 (27.0)
BV, *Candida* spp., C. glabrata, T. vaginalis	3 (0.2)	2 (0.1)	4 (0.3)
BV only	435 (31.9)	427 (28.6)	456 (30.8)
BV, *Candida* spp.	135 (9.9)	166 (11.1)	202 (13.6)
BV, C. glabrata	4 (0.3)	7 (0.5)	12 (0.8)
BV, T. vaginalis	71 (5.2)	108 (7.2)	80 (5.4)
BV, *Candida* spp., C. glabrata	8 (0.6)	8 (0.5)	8 (0.5)
BV, *Candida* spp., T. vaginalis	19 (1.4)	25 (1.7)	29 (2.0)
BV, C. glabrata, T. vaginalis	1 (0.1)	2 (0.1)	1 (0.1)
*Candida* spp. only	190 (13.9)	212 (14.2)	212 (14.3)
*Candida* spp., C. glabrata	22 (1.6)	13 (0.9)	15 (1.0)
*Candida* spp., T. vaginalis	12 (0.9)	15 (1.0)	16 (1.1)
*Candida* spp., C. glabrata, T. vaginalis	3 (0.2)	2 (0.1)	1 (0.1)
C. glabrata only	12 (0.9)	28 (1.9)	27 (1.8)
C. glabrata, T. vaginalis	0 (0.0)	1 (0.1)	0 (0.0)
T. vaginalis only	25 (1.8)	45 (3.0)	18 (1.2)

a *Candida* spp., *Candida* species group.

b The summary in each column includes only subjects with valid conclusive results for all four analytes.

### Prevalence and clinical performance.

BV, candidiasis, and trichomoniasis prevalences and the clinical performance of the investigational assays are shown in Table 3 by target for both clinician- and patient-collected samples. The overall prevalences of infection were similar for clinician- and patient collected vaginal swabs. The prevalence was highest for BV (49%), followed by candidiasis due to the *Candida* species group (29%).

**TABLE 3:** Overall investigational-assay performance

Target	Prevalence (%)	Specimen type (*n*)	Sensitivity		Specificity	
			No./total	% (95% CI)	No./total	% (95% CI)
BV	49.2	Clinician collected (1,413)	660/695^*a*^	95.0 (93.1–96.4)	643/718^*b*^	89.6 (87.1–91.6)
	49.3	Patient collected (1,405)	673/692^*c*^	97.3 (95.8–98.2)	612/713^*d*^	85.8 (83.1–88.2)
*Candida* sp. group	28.6	Clinician collected (1,485)	389/424	91.7 (88.7–94.0)	1,007/1,061	94.9 (93.4–96.1)
	28.6	Patient collected (1,477)	392/422	92.9 (90.0–95.0)	960/1,055	91.0 (89.1–92.6)
C. glabrata	4.0	Clinician collected (1,483)	50/59^*e*^	84.7 (73.5–91.8)	1,411/1,424^*f*^	99.1 (98.4–99.5)
	3.9	Patient collected (1,475)	50/58^*g*^	86.2 (75.1–92.8)	1,399/1,417^*h*^	98.7 (98.0–99.2)
T. vaginalis	9.9	Clinician collected (1,438)	137/142^*i*^	96.5 (92.0–98.5)	1,233/1,296^*j*^	95.1 (93.8–96.2)
	9.8	Patient collected (1,433)	136/140^*k*^	97.1 (92.9–98.9)	1,279/1,293^*l*^	98.9 (98.2–99.4)

a Of the 35 subjects with false-negative results, 10 subjects were Nugent intermediates and had BV infection status determined by modified Amsel criteria, and 15 were negative by modified Amsel criteria.

b Of the 75 subjects with false-positive results, 46 subjects were Nugent intermediates and had BV infection status determined by modified Amsel criteria, and 6 were positive by modified Amsel criteria.

c Of the 19 subjects with false-negative results, 6 subjects were Nugent intermediates and had BV infection status determined by modified Amsel criteria, and 7 were negative by modified Amsel criteria.

d Of the 101 subjects with false-positive results, 55 subjects were Nugent intermediates and had BV infection status determined by modified Amsel criteria, and 9 were positive by modified Amsel criteria.

e All 9 samples with false-negative results showed no growth of C. glabrata on chromogenic agar.

f Of the 13 samples with false-positive results, 2 showed high (4+) growth, 2 showed low (≤2+) growth, and 9 showed no growth of C. glabrata on chromogenic agar.

g Of the 8 samples with false-negative results, 7 showed no growth and 1 showed high (4+) growth of C. glabrata on chromogenic agar.

h Of the 18 samples with false-positive results, 2 showed high (4+) growth, 2 showed low (≤2+) growth, and 14 showed no growth of C. glabrata on chromogenic agar.

i Of the 5 samples with false-negative results, 3 were confirmed negative with the Aptima Trichomonas vaginalis assay.

j Of the 63 samples with false-positive results, 56 were confirmed positive with the Aptima Trichomonas vaginalis assay.

k Of the 4 samples with false-negative results, 3 were confirmed negative with the Aptima Trichomonas vaginalis assay.

l Of the 14 samples with false-positive results, 8 were confirmed positive with the Aptima Trichomonas vaginalis assay.

For clinician-collected samples, sensitivities and specificities against reference method samples were 95.0% and 89.6% for BV, 91.7% and 94.9% for the *Candida* species group, 84.7% and 99.1% for C. glabrata, and 96.5% and 95.1% for T. vaginalis. For patient-collected samples, sensitivities and specificities against reference method samples were 97.3% and 85.8% for BV, 92.9% and 91.0% for the *Candida* species group, 86.2% and 98.7% for C. glabrata, and 97.1% and 98.9% for T. vaginalis.

**FIG 2:**
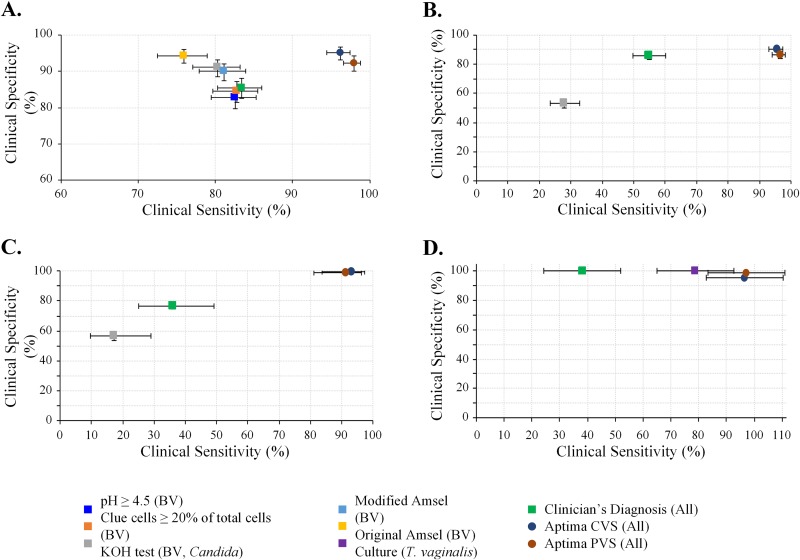
Comparison of clinical performance versus reference standards. Shown are paired clinical sensitivity and specificity estimates (with corresponding 95% confidence intervals [CI]) for Aptima results, clinician’s diagnoses, and clinical assessments compared to laboratory method diagnostic reference standards. (A) Detection of bacterial vaginosis compared to Nugent score. (B) Detection of *Candida* species group infection compared to culture. (C) Detection of C. glabrata infection compared to culture. (D) Detection of T. vaginalis compared to NAAT (Xpert TV assay). Note that some specificity error bars are too small to be visible. Original Amsel criteria were considered positive if at least 3 of the following were present: (i) clinician-reported signs of abnormal vaginal discharge that was thin and white, (ii) a pH of >4.5, (iii) clue cells comprising ≥20% of total cells, and (iv) a positive Whiff test. Modified Amsel criteria were considered positive if clue cells comprised ≥20% total cells and either pH was >4.5 or a whiff test was positive. CVS, clinician-collected vaginal swab; PVS, patient-collected vaginal swab.

The clinical sensitivities and specificities of clinicians’ diagnoses, in-clinic assessments, and the investigational assays were assessed compared to those of gold standard reference methods: Nugent score for BV, culture for VVC, and NAAT for trichomoniasis. Paired clinical sensitivity and specificity estimates for each target are shown in Fig. 2. Overall, the investigational assays had higher sensitivity and specificity than clinicians’ diagnoses and in-clinic assessments. For BV, sensitivity and specificity were ≥96.2% and ≥92.4%, respectively, for the investigational-assay samples, compared to 83.4% and 85.5% for clinicians’ diagnoses, 75.9% and 94.4% for original Amsel criteria, 81.1% and 90.1% for modified Amsel criteria, and ≤82.8% and ≤91.1% for any of the individual Amsel criterion components (vaginal pH, clue cells, and whiff test). For VVC due to the *Candida* species group or C. glabrata, sensitivity and specificity were ≥91.2% and ≥98.9%, respectively, for the investigational-assay samples compared to ≤27.9% and ≤56.4% for potassium hydroxide testing and ≤54.9% and ≤85.5% for clinicians’ diagnoses. For trichomoniasis, sensitivity was ≥96.4% for the investigational-assay samples compared to 78.8% for culture and 38.1% for clinicians’ diagnoses; specificity estimates were greater than 95% for all trichomoniasis detection methods. Table S1 in the supplemental material shows the positive and negative predictive values for the same comparisons; in general, the investigational assays were more predictive of infection than clinical assessments and clinicians’ diagnoses.

## DISCUSSION

Vaginitis affects millions of women annually and is the primary reason women in the United States visit their doctors (1). Women suffering from vaginitis are often underserved by the current paradigm of inaccurate or incomplete diagnosis guiding inadequate or inappropriate treatment. The resultant prolonged or recurrent vaginal dysbiosis impacts quality of life and has implications for preterm birth, acquisition/transmission of STIs and HIV, increased risk of pelvic inflammatory disease, and neonatal infections (21–29). Further, correct diagnosis of trichomoniasis is imperative in that sexual partners require treatment for control of this STI (6, 30, 31).

The investigational assays described here are FDA cleared for the differential diagnosis of BV, VVC, and trichomoniasis in symptomatic women; the tests are approved for use with both clinician-collected and patient-collected vaginal swabs. The results of these studies showed comparable sensitivities and specificities between clinician- and patient-collected samples for BV, VVC, VVC attributed to C. glabrata, and T. vaginalis. The investigational tests allow differential diagnoses of the 3 primary causes of vaginitis from a single vaginal swab rather than requiring the collection of multiple specimens to support microscopy, culture, KOH, and other methods. Specimen handling and contamination risk are minimized, with the swab placed directly into a sealed collection tube for testing on an automated instrument. Specimen integrity and stability for long-term storage are maintained by the stabilizing buffer within the collection tube.

Current in-clinic methods for diagnosing BV, VVC, and trichomoniasis present challenges that impact diagnostic accuracy and treatment. For BV, accurate diagnosis involves identifying the shift from a protective lactobacillus-dominated vaginal environment to pathogenic-anaerobe bacterial growth (32). Microbes indicated as causative agents in BV, such as *Gardnerella*, *Atopobium*, and *Prevotella*, are present in women both with and without BV as currently defined, so mere detection does not provide adequate specificity. The subjectivity of Nugent scoring of a Gram stain is apparent from the need for consensus scoring to improve accuracy, something that is not practical in clinical practice. Microscopy also presents challenges in terms of training, expertise, and equipment, and many clinicians have defaulted to empirical diagnosis and treatment, leading to incorrect management of infectious vaginitis. Multiplexed amplified molecular methods offer a viable alternative, where multiple microbes can be accurately quantitated at very high numbers (10^6^ CFU/ml or more), allowing the retraction of lactobacilli and overgrowth of *Gardnerella*, *Atopobium*, and other microbes in BV to be objectively measured, analyzed, and assessed. For diagnosis of candidiasis, culture is routinely performed, but it is time-consuming and labor-intensive and often lacks the sensitivity and species level identification of molecular methods (33, 34). Accurate identification of azole-resistant species, such as C. glabrata, is also important in guiding appropriate treatment. In the case of T. vaginalis, wet mounting must be performed within 10 min of sample collection while T. vaginalis is alive and motile; a positive wet-mount result requires organisms to be motile, not merely present.

Molecular methods are capable of overcoming many of these challenges, providing objective and reproducible results. Multiplex capabilities allow differential diagnoses for BV, VVC, and trichomoniasis unaffected by coinfection status. Concordance between the clinician- and patient-collected swabs for the investigational tests described here also indicate that these tests are robust against sample collection, preparation, and interpretation artifacts that can confound diagnoses. The patient-collected swab also allows the potential for patient comfort and convenience while streamlining clinical workflow.

Strengths of the study include the broad geographic U.S. distribution of enrolled subjects, ensuring a robust evaluation of the investigational tests in these populations. The use of multiple reference methods for all indications (for BV, Nugent plus Amsel to adjudicate Nugent intermediates; for VVC, 2 cultures plus PCR/bidirectional sequencing of positive cultures; for T. vaginalis, a composite of culture plus NAAT) improves accuracy and reduces potential bias toward any single metric.

Limitations include limited representation of certain ethnic groups, primarily Asians and Pacific Islanders (who were grouped with “other”). Molecular methods are also highly specific, potentially impacting the sensitivity to disease attributed to minor species (e.g., *Prevotella* and Candida krusei) not included in the assay design.

The availability of highly sensitive and specific NAATs for vaginitis that can be performed on automated analyzers are a welcome addition to previously available tests. The investigational molecular assays offer sensitive and specific detection of vaginitis and are capable of providing differential diagnoses of multiple etiologies from a single vaginal swab. In addition, the differentiation of C. glabrata from other species causing VVC may be helpful in guiding therapy for the infection, which is often azole resistant. These types of tests can provide objective tools for the clinician to accurately diagnose and treat patients.

## Supplementary Material

Supplemental file 1
