# Supplementary material for: Clinical Validation of the Aptima Bacterial Vaginosis and Aptima Candida/Trichomonas Vaginitis Assays: Results from a Prospective Multicenter Clinical Study
Source: J Clin Microbiol. 2020 Jan 28;58(2):e01643-19. doi: 10.1128/JCM.01643-19 (PMC6989072; doi:10.1128/JCM.01643-19)
Supplement: Supplemental file 1 [file JCM.01643-19-s0001.pdf]

Supplemental

Clinical validation of the Aptima Bacterial Vaginosis and Aptima Candida/Trichomonas Vaginitis Assays: results from a prospective multi-center clinical study

Jane R. Schwebke MD[a], Stephanie N. Taylor MD[b], Ronald Ackerman MD[c], Robert Schlaberg MD MPH[d], Neil Quigley PhD[e], Charlotte A. Gaydos DrPh[f], Steven E. Chavoustie MD[g], Paul Nyirjesy MD[h], Carmelle V. Remillard PhD[i], Philip Estes BS[i], Byron McKinney BS[i], Damon K. Getman PhD[i] Craig Clark, PhD[i]

Supplemental Table 1. Performance of clinical evaluation criteria and clinician diagnosis: Predictive values

	PPV	NPV
	% (95% CI)	
**Detection of BV relative to Nugent Score**		
pH >4.5	83.9 (81.4- 86.2)	81.4 (78.8- 84.0)
Clue cells ≥20% total cells	85.2 (82.8- 87.5)	81.9 (79.3- 84.4)
Whiff test	90.7 (88.4- 92.7)	81.1 (78.6- 83.4)
Original Amsel[a]	93.6 (91.4- 95.4)	78.4 (76.0- 80.7)
Modified Amsel[bc]	89.8 (87.5- 91.9)	81.5 (79.1- 83.9)
Clinician's diagnosis	86.1 (83.7- 88.4)	82.7 (80.1- 85.1)
Aptima BV assay (CVS)	95.6 (93.9- 96.9)	95.9 (94.1- 97.2)
Aptima BV assay (PVS)	93.3 (91.4- 94.9)	97.7 (96.3- 98.7)
**Detection of *Candida* species group relative to culture**		
KOH test	15.0 (12.8-17.4)	71.1 (69.4-72.9)
Clinician's diagnosis	53.1 (48.8-57.3)	86.4 (85.0-87.8)
Aptima CV/TV assay (CVS)	74.3 (70.9-77.6)	98.6 (97.7-99.2)
Aptima CV/TV assay (PVS)	67.8 (64.6-71.0)	98.8 (98.0-99.4)
**Detection of *C. glabrata* relative to culture**		
KOH test	1.6 (0.8-2.6)	94.4 (93.8-95.2)
Clinician's diagnosis	5.9 (4.0-7.9)	96.8 (96.2-97.4)
Aptima CV/TV assay (CVS)	85.7 (76.0-92.6)	99.7 (99.3-99.9)
Aptima CV/TV assay (PVS)	76.5 (66.8-84.7)	99.6 (99.2-99.9)
**Detection of *T. vaginalis* relative to reference NAAT**		
Culture	99.1 (95.4-100.0)	97.8 (97.1-98.5)
Clinician's diagnosis	94.6 (86.3-98.7)	94.0 (93.3-94.8)
Aptima CV/TV assay (CVS)	67.7 (62.3-73.0)	99.6 (99.1-99.9)
Aptima CV/TV assay (PVS)	89.9 (84.6-93.9)	99.7 (99.3-99.9)

CVS = clinician-collected swab, PVS = patient-collected swab

[a] Positive if at least 3 of the following: 1) clinician-reported signs of abnormal vaginal discharge that is thin and white; 2) pH > 4.5; 3) clue cells ≥20% total cells' 4) positive Whiff test.

[b] Positive if clue cells ≥20% total cells and either pH > 4.5 or positive Whiff test.
